# Bologna Healing Stifle Injury Index: A Comparison of Three Surgical Techniques for the Treatment of Cranial Cruciate Ligament Rupture in Dogs

**DOI:** 10.3389/fvets.2020.567473

**Published:** 2020-10-28

**Authors:** Stefania Pinna, Francesco Lanzi, Lisa Grassato

**Affiliations:** Department of Veterinary Medical Sciences, University of Bologna, Bologna, Italy

**Keywords:** cruciate cranial ligament, questionnaire, BHSII, surgical treatment, dog

## Abstract

The aim of this retrospective study was to test the efficacy of the Bologna Healing Stifle Injury Index (BHSII) in assessing the medium-term outcomes of dogs treated for cranial cruciate ligament rupture. This tool can be used for comparison across surgical interventions. The study population included 53 dogs with unilateral cranial cruciate ligament rupture treated using either Paatsama, Tight-Rope or tibial tuberosity advancement techniques, and 20 orthopedically sound dogs for comparative purposes. The BHSII was utilized for all the treated dogs at the time of surgery, and 1, 3, and 6 months postoperatively, while it was utilized twice in the control group. Although all the techniques achieved a successful outcome at the end of the evaluation, the application of the BHSII permitted differentiating results at each time point and stimulating discussion regarding the rapidity and degree of the healing process for each technique. It also pointed out some incongruities between the owner's and the clinician's assessment of the process. These achievements demonstrated that the BHSII should be considered by the research and clinical communities as an effective and easy tool which can be used as a repeatable and standardized method of comparison of the progress at different time points toward a final good outcome in dogs treated for cranial cruciate ligament rupture.

## Introduction

Rupture of the cranial cruciate ligament (CCL) is a common canine orthopedic injury, and a frequent cause of pain and lameness (1, 2). Surgical treatment is suggested to restore joint function and improve the quality of life (3). Over the years, many surgical procedures have been reported, and several studies have been carried out to compare the outcomes among techniques. However, none of these techniques has been considered superior to the others (4–6). The studies evaluated subjective and objective findings, including owner impression, clinical examination, radiographic evaluation, synovial fluid analysis, macro and microscopic features, gait analysis and biomechanical testing. Gait analysis is probably the most objective and repeatable test, but it requires expensive equipment and specific training that not all clinicians can afford (7, 8).

Evidence-based medicine has been defined as the conscientious, explicit and judicious use of the current best evidence in making decisions regarding the care of individual patients (9). The next step was the adaption of this discipline to veterinary medicine; Evidence-based veterinary medicine provides a tool for recognizing the best choice among various treatments (4, 10, 11). The Bologna healing stifle injury index (BHSII), a tool for evaluating the healing process of stifle joints treated for CCL rupture, has recently been validated (12). This is a complete tool obtained from the combination of clinician and owner assessments, with subjective and objective evaluations, useful for guiding clinical practitioners in their clinical decision-making. This tool has already been proven to be useful in understanding the progression of the outcome reported in dogs treated for CCL rupture with a specific surgical procedure, such as biceps femoris muscle transposition and the intracapsular mini-TightRope system at different time points (13, 14). It is the Authors' belief that it can also be demonstrated to be a helpful tool in comparing different techniques during the healing progress and the return to the expected good quality of life of the dogs.

Many surgical approaches have been suggested for the treatment of CCL rupture, and they are classified as intracapsular, extracapsular, and osteotomy procedures (5). The purpose of the present study was to determine whether the BHSII could detect changes in outcome in dogs treated for CCL rupture with different techniques. The hypothesis was that the BHSII had the ability to reveal significant differences in times of healing when different procedures, such as Paatsama (PAATS), Tight-Rope (TR), and tibial tuberosity advancement (TTA) were utilized.

## Materials and Methods

The medical records of dogs with unilateral CCL rupture were obtained from the archive of the University Hospital of the Department of Veterinary Medical Sciences, University of Bologna, Italy, and they were retrospectively reviewed.

The dogs in the present study were divided into two groups: a surgical group and a control group. The Surgical Group included dogs which had undergone one of the three above-mentioned surgical techniques: the Paatsama (PAATS Group), Tight-Rope (TR Group), or tibial tuberosity advancement (TTA Group) techniques. The inclusion criteria were that the medical records included a complete BHSII registered at the time of surgery (T0) and at 1, 3, and 6 months after surgery, indicated as T1, T3, and T6, respectively. Medium-size dogs of both genders, all ages and medium size breeds were included. Each medical record also included the written informed consent of the owners. The exclusion criteria were dogs with an incomplete BHSII and those with serious complications requiring a second surgery. The Control Group included orthopedically sound dogs for comparative purposes. In these cases, the BHSII was completed at a first examination (T0) and 15 days later (T15).

All the dogs in this study were chosen from those previously evaluated for the validation of the BHSII tool (12). The BHSII is composed of two parts: a survey for dog owners (BHSII-OQ: Owner Questionnaire) and an orthopedic examination performed by the veterinarian (BHSII-CR: Clinical Record). The BHSII-OQ is made up of three domains: pain (*P* = 12 questions), stiffness (S = 5 questions), and function (F = 7 questions). The BHSII-CR is made up of two domains: visual examination (V = 3 questions) and manual examination (M = 7 questions). Each question, for a total of 34 questions or items, has a multiple choice answer (0–4), and the sum of the total score of each domain of both the BHSII-OQ and the BHSII-CR were normalized, resulting in a scale of values (100-0) in which 100 indicates the absence of problems and 0 the presence of extreme symptoms (see https://www.frontiersin.org/articles/10.3389/fvets.2019.00065/full#supplementary-material)(12).

### Statistical Analysis

The descriptive statistics were calculated, and normal distribution was assessed using the Kolgomorov-Smirnov test. Continuous data (age, body weight, and the BHSII, BHSII-OQ and BHSII-CR scores of the dogs) were expressed as mean and standard deviation (SD), and categorical data (gender, breed and affected limb) were expressed as frequencies.

The Kolgomorov-Smirnov test rejected normality only in the Control Group, and the Wilcoxon test was used to compare the BHSII scores between the two evaluations. The Mann-Whitney test was used to compare the scores between the Control Group and the Surgical Group.

Repeated Measures analysis of variance (ANOVA) was used to compare the BHSII scores collected at the four time points, at the time of surgery and after treatment in each group treated, and to compare the changes in the BHSII scores between the groups at the same time point; the Bonferroni correction was used as a *post-hoc* analysis.

All variations of the scores were expressed as a percentage, and they were compared to the previous measurement at each time point.

Repeated measures ANOVA was also carried out to assess the changes of each domain score during the follow-up in each group treated.

The data were analyzed using a statistical software program (MedCalcR Software 16.8.4, Ostend, Belgium). Significance for all the analyses was set at *P* < 0.05.

## Results

In this study, 73 dogs of different breeds were included. There were 23 males and 50 females, the mean age was 5.6 ± 2.8 years and the mean body weight was 29.8 ± 10.1 kg; the stifle joints involved were 45 right and 28 left stifles. Fifty-three dogs (Surgical Group) presented with CCL rupture and were divided into three groups based on the surgical procedure they underwent: PAATS Group (*n* = 12), TR Group (*n* = 16) and TTA Group (*n* = 25). In addition, the Control Group included 20 dogs. The data regarding the descriptive statistics are listed in Table 1.

**Table 1:** Epidemiologic data of dogs.

	**PAATS**	**TR**	**TTA**	**Control**
	**(*n*. 12)**	**(*n*. 16)**	**(*n*. 25)**	**(*n*. 20)**
**Gender**				
Male (*n*.)	6	7	6	4
Female (*n*.)	6	9	19	16
**Age (months)**				
Mean ± SD	5 ± 2.9	6.3 ± 3	5.4 ± 2.3	5.7 ± 3.3
**Weight (kg)**				
Mean ± SD	36.8 ± 11.1	29.6 ± 12.4	29.2 ± 7.7	26.7 ± 8.5
**Limb**				
Right (*n*.)	6	9	10	20
Left (*n*.)	6	7	15	0
**Breed (*****n*****.)**				
Cross-Breed	3	8	6	3
Amstaff	1		5	
Labrador retriever	4	2	5	1
Golden retriever			1	6
Beagle			2	
Boxer	1	1	3	
Cane corso	2			
Rottweiler		2		
Border collie		2	1	3
Others	1	1	2	7

*Control, dogs without orthopedic diseases; PAATS, Paatsama; TR, TightRope; TTA, Tibial Tuberosity Advancement techniques*.

In the Control Group there were no changes in the BHSII, the BHSII-CR and the BHSII-OQ scores between two consecutive evaluations (*P* = 0.16; *P* = 0.33; *P* = 0.20, respectively), but there were significant differences when the BHSII, BHSII-CR and BHSII-OQ scores of the Control Group at T0 and T15 were compared to the Surgical Group at T0 and the first evaluation after treatment (T1) (*P* < 0.0001). The assessments were statistically significant even when the Control Group was compared to each group treated.

**Figure 1:**
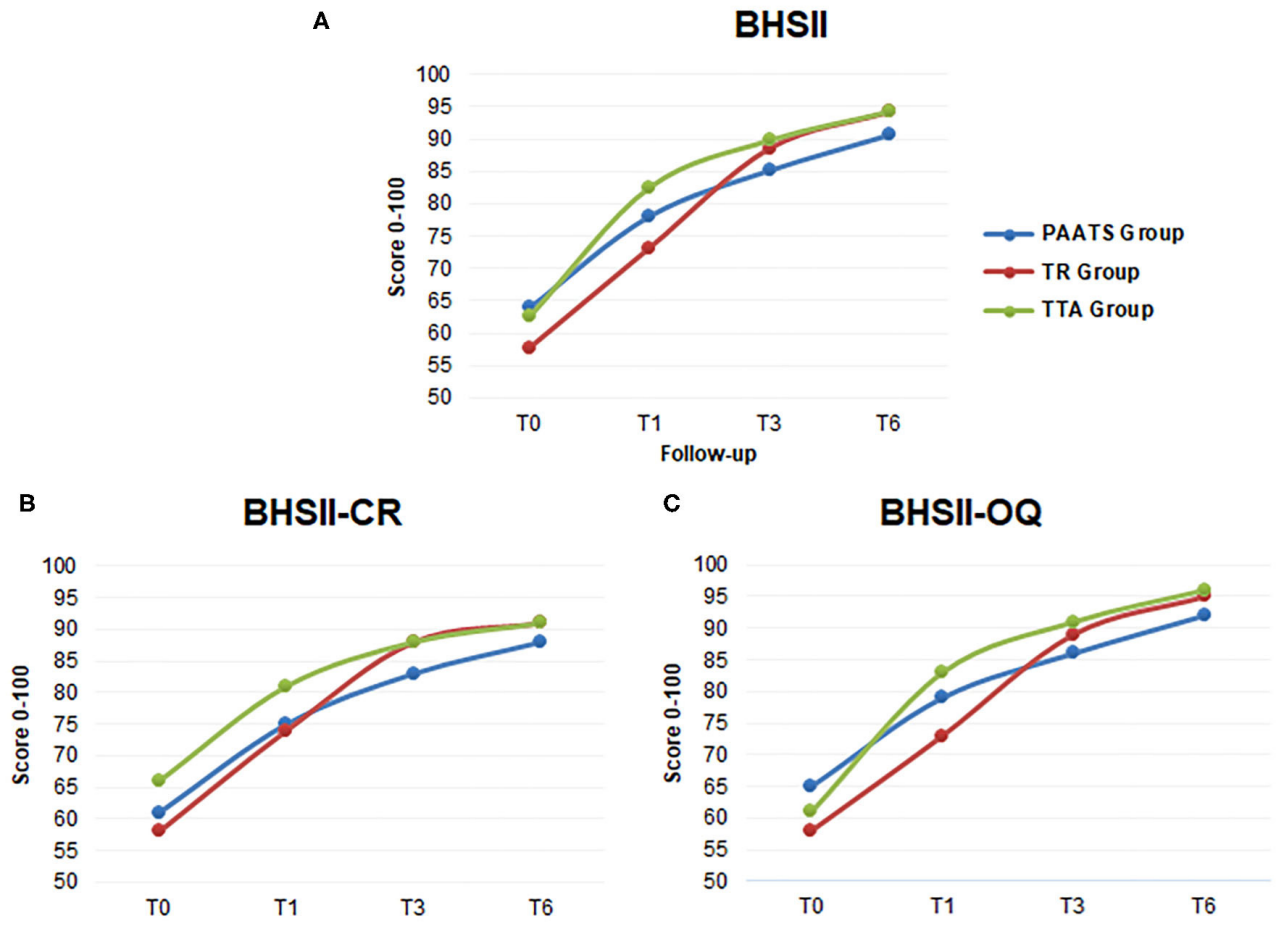
The line charts show the mean scores for each surgical group during follow-up. **(A)** The lines give information regarding the positive changes in the BHSII over time. **(B)** The graph shows the mean scores of the Clinical Records. **(C)** The graph is the elaboration of the Owner Questionnaires. T0: time at surgery, T1, T3, and T6: 1, 3, and 6 months after surgery, respectively. The blue line: group treated with the Paatsama technique, the red line: the TightRope technique and the green line: the Tibial tuberosity advancement technique.

In the Surgical Group, a repeated measures ANOVA of the BHSII score revealed a statistically significant improvement at each time point. The same significant results were obtained from the BHSII-CR and the BHSII-OQ evaluations. The changes in the BHSII scores were statistically significant at each time point in each of the three groups (PAATS, TR, TTA) when compared to baseline T0. In the PAATS Group, the evaluation of the BHSII-CR revealed a non-significant difference (*P* = 0.21) between T1 and T3. The improvement between T3 and T6 in the BHSII-CR was not significant in any group (PAATS group, *P* = 0.22; TR Group, *P* = 0.07; TTA Group, *P* = 0.48). In the PAATS Group, the evaluation of the BHSII-OQ also presented a non-significant *P*-value (*P* = 0.06) between T1 and T3. All the other BHSII-OQ investigations were statistically significant in each group from T0 to T6. All the data are listed in Table 2, and the trend of the improvements is graphically reported in Figure 1. The analyses carried out among the three groups treated did not show any statistically significant differences.

**Table 2:** Intragroup evaluation.

**Time**	**Groups (*n*. dogs)**	**BHSII**	**BHSII-CR**	**BHSII-OQ**
T0	Control (*n*. 20)	97.9 ± 2.5	98.4 ± 2.5	97.8 ± 3.2
T15		98.2 ± 2.6^*^	98.6 ± 2.4^*^	98.1 ± 3.5^*^
T0	PAATS (*n*. 12)	63.9 ± 10.9	60.6 ± 8.4	65.4 ± 14.4
	TR (*n*. 16)	57.8 ± 8.8	57.8 ± 10.5	57.8 ± 12.4
	TTA (*n*. 25)	62.6 ± 15.1	66.3 ± 8.5	61.3 ± 19.2
T1	PAATS (*n*. 12)	78.1 ± 9.1	74.7 ± 7.4	79.4 ± 11.9
	TR (*n*. 16)	73.2 ± 15.5	74.4 ± 10.1	72.7 ± 19.6
	TTA (*n*. 25)	82.5 ± 11.9	81.4 ± 8.5	82.9 ± 15.3
T3	PAATS (*n*. 12)	85.1 ± 8.5	82.7 ± 11.3^*^	86.1 ± 9.7^*^
	TR (*n*. 16)	88.6 ± 6.8	87.5 ± 4.1	88.9 ± 8.9
	TTA (*n*. 25)	89.8 ± 7.3	87.6 ± 7.3	90.6 ± 9.6
T6	PAATS (*n*. 12)	90.6 ± 6.4	88.3 ± 6.7^*^	91.6 ± 7.7
	TR (*n*. 16)	94.2 ± 4.2	91.3 ± 6.8^*^	95.2 ± 3.9
	TTA (*n*. 25)	94.2 ± 5.1	90.7 ± 5.8^*^	95.5 ± 5.6

*Mean (± SD) scores calculated for each group during the follow-up. The asterisk indicates the time at which the improvement was not significant when one score was compared to a previous score*.

*BHSII, Bologna Healing Stifle Injury Index; BHSII-OQ, BHSII-Owner Questionnaire; BHSII-CR, BHSII-Clinical Record; T0, preoperative time/first evaluation; T15, (15 days) second evaluation for the Control group; T1, 1 month after surgery; T3 and T6, 3 and 6 months after surgery*.

* *P-value was not significant. The scores are expressed as mean ± SD (Standard Deviation)*.

**Figure 2:**
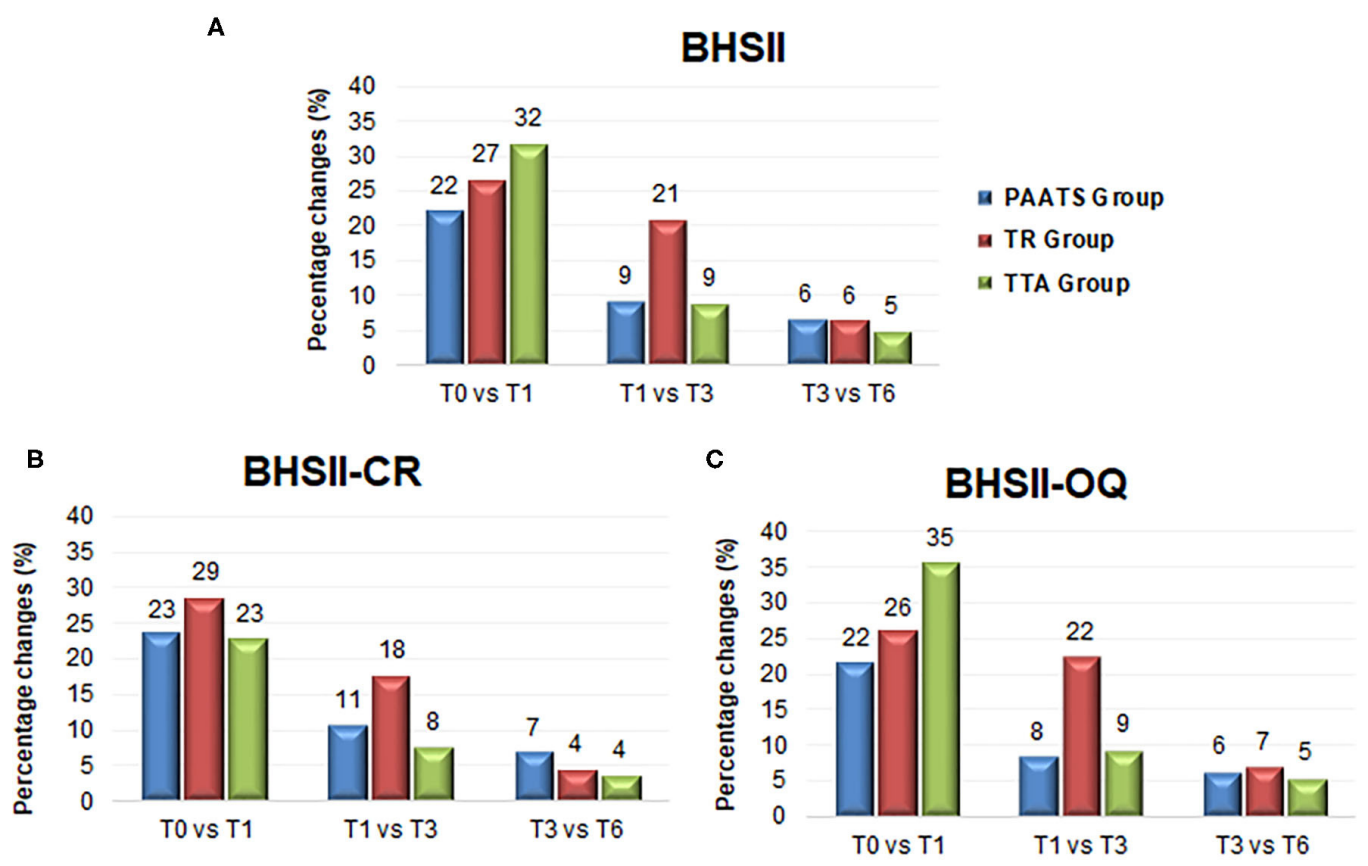
The bar charts show the percentage of change in the mean scores assessed at each time of follow-up as compared with the next time point. **(A)** The graph reveals the positive changes in the BHSII scores for each surgical group. **(B)** The graph shows the percentage of change in the Clinical Records. **(C)** Owner Questionnaires. T0: time before surgery, T1, T3, and T6: 1, 3, and 6 months after surgery, respectively. vs: versus. The blue bar: the group treated with the Paatsama technique, the red bar: the TightRope technique and the green bar: the tibial tuberosity advancement technique.

The analyses regarding the modification of the BHSII scores revealed that the greatest changes in percentage occurred between T0 and T1 in each group, with emphasis on the TTA Group which increased by 32% as compared to its baseline while the increases were of only 9 and 5% from T1 to T3, and from T3 to T6, respectively. The smallest increase percentage from T0 to T1 was recorded in the PAATS Group (22%). The TR Group showed an evident increase percentage at T1 and between T1 and T3 (27 and 21%, respectively) (Figure 2). The percentage of change in the BHSII-CR and the BHSII-OQ were also compared between two consecutive evaluations. In PAATS Group, the changes in the clinical examination scores (BHSII-CR) were high at T1 (23%), increasing without a peak after 3 and 6 months (11 and 7%, respectively). The BHSII-OQ score already improved at T1 (22%). The TR Group showed a continuous percentage increase in the BHSII-CR at each time point (29, 18, and 4%, at T1, T3, and T6, respectively); the same trend was true for the BHSII-OQ. The TTA Group reported a BHSII score similar to those in both the BHSII-CR and the BHSII-OQ, with a peak of improvement at T1 (32, 23, and 35%, respectively) as compared to T3 (9, 8, and 9%, respectively) (Figure 2).

**Figure 3:**
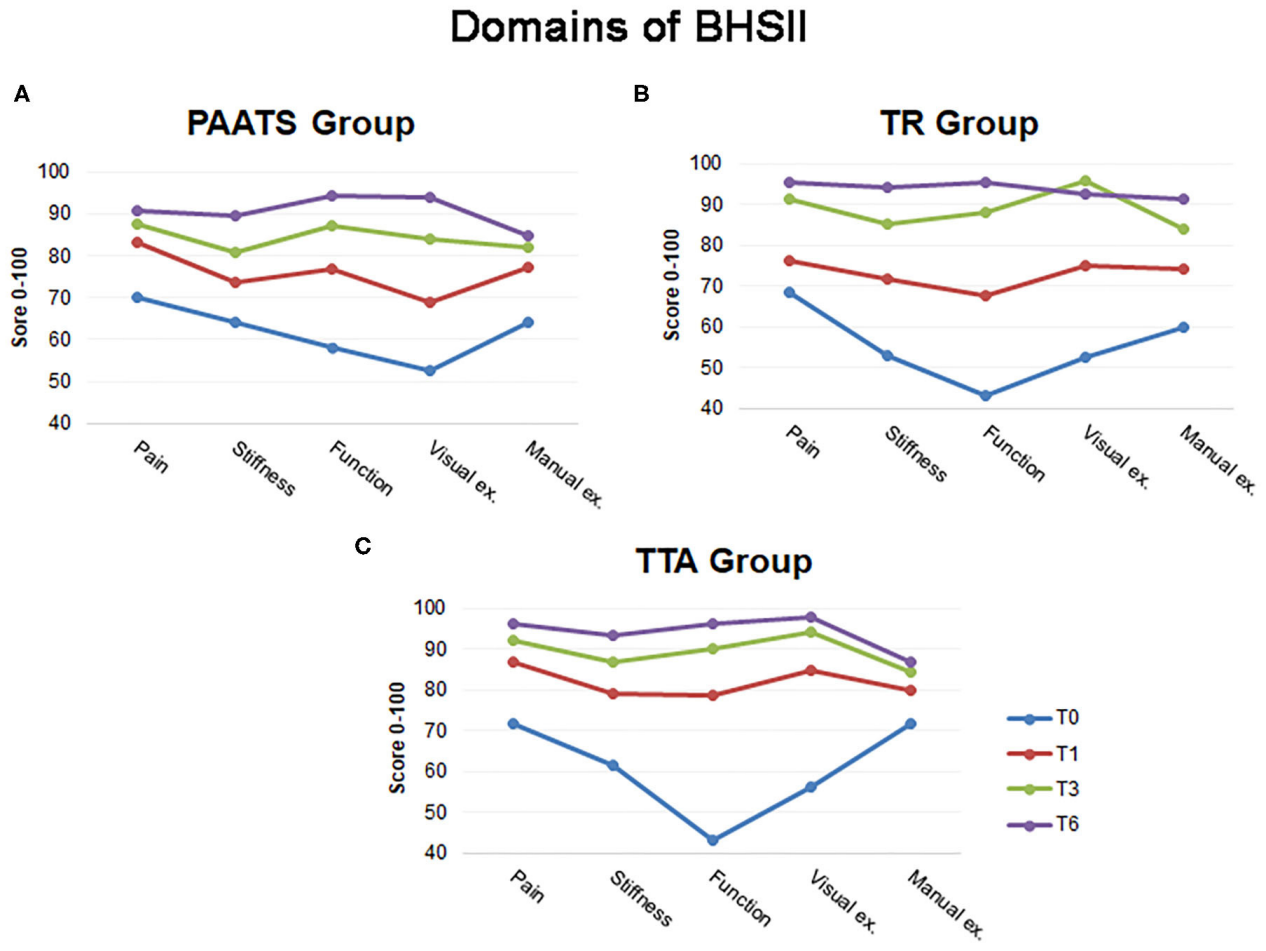
The illustration shows the mean scores for each domain of the BHSII during follow-up. **(A)** The lines give information regarding the positive changes in the BHSII evaluated for the Paatsama group, **(B)** for the TightRope group and **(C)** for the Tibial Tuberosity Advancement group. The blue line: T0: time before surgery, the red line: T1: 1 month after surgery, the green line: T3: 3 months after surgery and the violet line: T6: 6 months after surgery.

The scores obtained from the analyses of each domain for each surgical treatment showed a continuously significant improvement when compared to the baseline T0. However, repeated measures ANOVA revealed various, not statistically significant, changes (*P* > 0.05) at different time points when compared to the previous time point (Table 3 and Figure 3).

**Table 3:** Mean (± SD) scores of the domains evaluated for each surgical group during the follow-up.

**Time**		**BHSII-OQ**			**BHSII-CR**	
		**Pain**	**Stiffness**	**Function**	**Visual ex.**	**Manual ex.**
T0	PAATS	70.1 ± 4.4	64.2 ± 20.1	58.0 ± 22.4	52.8 ± 14.4	64.0 ± 11.6
	TR	68.4 ± 14.8	52.8 ± 23.7	43.3 ± 17.9	52.6 ± 11.7	60.0 ± 12.8
	TTA	71.7 ± 20.2	61.4 ± 25.7	43.3 ± 21.2	56.0 ± 11.2	71.5 ± 11.1
T1	PAATS	83.3 ± 9.9	73.8 ± 20.6^*^	76.8 ± 14.2^*^	68.8 ± 11.3	77.4 ± 9.8
	TR	76.2 ± 18.8^*^	71.6 ± 24.5	67.6 ± 27.7	75.0 ± 18.0	74.1 ± 9.4
	TTA	86.9 ± 13.8	79.2 ± 23.0	78.7 ± 18.3	84.7 ± 14.8	79.8 ± 8.6
T3	PAATS	87.7 ± 10.1	80.8 ± 14.6^*^	87.2 ± 9.7^*^	84.0 ± 17.9	82.1 ± 11.8^*^
	TR	91.1 ± 8.6	85.3 ± 13.1	87.9 ± 11.7	95.8 ± 7.5^*^	83.9 ± 3.9
	TTA	92.3 ± 9.6^*^	87.0 ± 14.0^*^	90.0 ± 11.0	94.3 ± 10.1^*^	84.2 ± 8.7^*^
T6	PAATS	90.8 ± 10.7^*^	89.6 ± 10.1	94.3 ± 5.4	93.8 ± 10.7	84.8 ± 7.9^*^
	TR	95.6 ± 4.2^*^	94.1 ± 6.4^*^	95.5 ± 5.6^*^	92.7 ± 12.5^*^	91.3 ± 6.8
	TTA	96.1 ± 5.8^*^	93.2 ± 9.6^*^	96.1 ± 7.3	98.0 ± 5.5^*^	87.0 ± 7.7^*^

*The asterisk indicates the time at which the improvement was not significant when one score was compared to the previous score*.

*BHSII-OQ, BHSII-Owner Questionnaire; BHSII-CR, BHSII-Clinical Record*.

* *Not significant*.

## Discussion

The objective of this study was to assess whether the BHSII had the ability to determine the differences in recovery time regarding the different techniques.

The BHSII was previously validated on a large sample of dogs without considering the type of surgery performed (12); consequently, the present study represented an additional step in applying the tool in groups of dogs treated with three different techniques. The goal was not to achieve a classification regarding the best surgery, but to evaluate the progression of the outcome in the clinical assessment (i.e., lameness, presence of pain, presence of crepitus, variations in range of motion, quality of life) in different postoperative time points, and to demonstrate that the BHSII represented a novel and useful tool for comparisons. The results confirmed the effectiveness of the tool in detecting the clinical progress of dogs treated for CCL rupture.

The choice of the Paatsama, TightRope and TTA techniques reflected the three possible different approaches for treating a CCL rupture in the stifle joint (intracapsular, extracapsular, and osteotomy); these approaches could have different impacts regarding morbidity and, consequently, the quality of life (3). The choice of these three surgical techniques, rather than other common ones, was related to the inclusion criteria. For instance, with regard to the BHSII completed up to 6 months after surgery, there was difficulty getting feedback from the owners of dogs treated with TPLO when there was an absence of clinical alterations. Instead, the owners of dogs undergoing the TTA technique were motivated to have their dogs re-checked for clinical healing and complete mineralization within the osteotomy gap.

The results obtained could have been influenced by both the choice of the surgical procedure and the exclusion of dogs with serious complications requiring a second surgery. In the first case, the surgical choice was dependent on surgeon experience. This represents a limitation of the study and a future a randomized trial would be interesting to avoid this weakness and to evaluate the validity of the findings of the present paper when applying the tool to different techniques. It is the Authors' belief that this paper represents a good starting point to describe the potential of the tool to be applied for intra- and inter-groups evaluation and a prospective randomized study would be useful to confirm these findings. In the second case, the BHSII scores of dogs with major complications would have provided a decrease in the outcomes with obvious misinterpretation of the mean values.

Another limitation of the study was that the radiographic examination of the affected joint was not included and possible correlations between radiographic findings and the results of utilizing the tool were not studied. As explained in a previous article, the radiographic assessment of osteoarthritis was not included in the BHSII tool because the signs of osteoarthritis, and their respective radiographic scores, only worsen or stabilize, but not improve, over time (12, 15). It would be interesting to relate the BHSII results to osteoarthritis radiographic findings, despite the fact that the functional and the radiographic appearances of the limb do not always correlate (15, 16); therefore, other studies from this perspective are suggested.

Another limitation of this study was the absence of a control group of dogs with untreated CCL rupture. It is not ethical not to treat an animal with pain and evidence of lameness according to the Italian ethical committee. For this reason, the Control Group was made up of orthopedically sound dogs to confirm the reliability and responsiveness of the BHSII (12). Although small dogs with CCL injury could be treated conservatively (17) the Control Group in the present study was composed of medium dogs to keep the weight of the population uniform. Even though the BHSII can be applied to small dogs (13, 14), the inclusion criteria specifying medium size dogs was intended to reduce weight variability as compared to the previous study in which all weights were included (12).

The Authors are aware of the intrinsic limitations of the retrospective studies and the present one is meant to be a demonstration of the potential of the BHSII to be used as a useful tool in the comparison of the clinical progress deriving from the application of different surgical techniques. BHSII scoring system has already been validated in a previous study; the intention of the present manuscript was to demonstrate that it could easily be used to compare different surgical techniques, and to extrapolate important and easy-to-read information. Future prospective studies are suggested to demonstrate whether the encouraging findings showed by this study remain valid on a randomized trial and to eliminate the limitations led by its retrospective nature.

The outcomes obtained from the analyses conduced regarding each technique indicated that the BHSII scores were statistically significant at each time point. All the surgical techniques led to a successful outcome, but at different times and with a different trend between clinical evaluation and owner opinion. As expected, a change in the BHSII score was a useful indicator for determining the efficacy of the surgical treatment for CCL rupture. Many objective and subjective parameters have influenced the evaluation of the outcome of the intervention, as was evident in the BHSII-CR. For all the techniques, the scores recorded by the BHSII-CR 3 months after surgery (T3) continued increasing, albeit slowly, and were not statistically significant between T3 and T6. Instead, the owners' opinions (BHSII-OQ), which were part of the complete picture of the healing process, perceived the well-being of their dogs to be statistically significant until the end of the study (3, 12).

Interestingly, the trend reported by the PAATS Group was unusual. The scores of both the BHSII-CR and the BHSII-OQ revealed a significant improvement only in the first month after surgery; the progression toward a final good outcome was then slow and, at T3, it was not statistically significant (Table 2). This trend could be due to the morbidity of the intracapsular procedure in which open arthrotomy and bone tunnels were carried out (18, 19). This finding was also supported by the percentage of change in the BHSII which showed the main improvement in the first month (Figure 2), and by the assessment of the stiffness, function and manual examination domains which had a *P* > 0.05 at T1 (Table 3). These outcomes could have been indicative of the greater effort of the dogs treated with the Paatsama technique to reach complete recovery.

In the TR Group, the percentage of change in the BHSII score was high from T1 to T3 as compared to the other techniques, which revealed the need for more time to arrive at high score values (Figure 2). An explanation for this tendency could be found in the assessment of the items of the BHSII. The pain score, which is included in the BHSII-OQ, improved at T1 but was not statistically significant until T3, and the visual examination assessment was also indicative of a slow increase (Table 3). The range of motion, evaluated in the manual examination of the BHSII-CR, may have involved stiffness which influenced the functionality of the limb (12, 20, 21). The BHSII-OQ also had a domain concerning stiffness which reported a continuous change until T6; this was indicative of a behavior perceived as continuous improvement by the owner. In fact, the trend of both the BHSII-CR and the BHSII-OQ was similar.

Analyses of the TTA Group revealed a process of healing completed in a shorter time when compared to the other techniques. In this group, the BHSII improved quickly, namely 32% from T0 to T1, and four domains out of five had already reached a high score at T1. Additional score changes were no longer significant at T3 (Table 3).

The statistical comparison between the techniques at the same time point did not reveal significant differences All the dogs achieved full recovery at 6 months; however, some slight differences in the progress toward the final good outcome were detected, as is clearly illustrated in the graphs of Figure 1.

Since the initial scores were not the same for all the dogs at T0, the percentage of change was calculated at every time point based on the previous observation in order to obtain the best elaboration of the trend of the various techniques (Figure 2). At T1, the TTA Group recorded the highest percentage, followed by the TR Group and then by the PAATS Group. At T3, the TR Group showed an increase in percentage greater than the other groups; this was presumably due to owner opinion regarding the low invasiveness of the TightRope technique.

The results of the present paper were in contrast with the outcomes described by Christopher et al. (22) who reported that, even if both the TTA and TR procedures returned the stifle joint to full function, the TR led to better results as compared to the TTA (22). In the present study the Authors showed that the TTA Group achieved more rapid healing, not only with regard to what was observed by the surgeon according to the clinical records but also with regard to the owner's opinion. It is possible that the exclusion of some cases with major complications (requiring revision surgery), even if this number was very low, could have had an influence on this hypothesis. This type of exclusion regarded all the techniques considered in this study; it is the Authors' belief that this did not represent a great bias.

Various studies have been published which have compared the surgical techniques for treating CCL rupture. Over time, lateral suture stabilization, intracapsular over-the-top and TPLO, TTA and TightRope, have been evaluated (7, 20, 23). These papers have reported evaluation or outcome assessment using force plate analysis, surgeon evaluation and pet owner subjective evaluation. It is not possible to make a direct comparison with other outcomes when the methods of measurement are different. This study attempted to establish uniform evaluation methods and measurement criteria.

The aim of the Authors was to use the BHSII to monitor the clinical outcome and progress after stifle joint surgical treatment in order to improve decision-making, rational diagnostics and treatments. The findings in the present study confirmed the ability of the BHSII to reveal differences in recovery time in dogs treated for CCL rupture.

## Data Availability Statement

All datasets presented in this study are included in the article/supplementary material.

## Ethics Statement

Ethical review and approval was not required for the animal study because this is a retrospective study with data collection of routine clinical investigations at the university teaching hospital, and the approval of the ethics committee is not required. Written informed consent was obtained from the owners for the participation of their animals in this study.

## Author Contributions

SP: conceptualization, study design, data elaboration, manuscript writing and editing, and statistical analyses. FL: data collection, manuscript editing, and clinical evaluations. LG: data collection, manuscript writing, and clinical evaluations. All authors have revised and discussed the manuscript, read, and approved the final version of the manuscript for publication.

## Conflict of Interest

The authors declare that the research was conducted in the absence of any commercial or financial relationships that could be construed as a potential conflict of interest.
